# Biophysical characterization and a roadmap towards the NMR solution structure of G0S2, a key enzyme in non-alcoholic fatty liver disease

**DOI:** 10.1371/journal.pone.0249164

**Published:** 2021-07-14

**Authors:** Michael W. Moran, Elizabeth P. Ramirez, James D. Zook, Alicia M. Saarinen, Bobby Baravati, Matthew R. Goode, Vasiliki Laloudakis, Emily K. Kaschner, Tien L. Olson, Felicia M. Craciunescu, Debra T. Hansen, Jun Liu, Petra Fromme

**Affiliations:** 1 Biodesign Center for Applied Structural Discovery, Arizona State University, Tempe, AZ, United States of America; 2 School of Molecular Sciences, Arizona State University, Tempe, AZ, United States of America; 3 Department of Biochemistry and Molecular Biology, Mayo Clinic in Arizona Scottsdale, AZ, United States of America; 4 Department of Cardiovascular Medicine, Mayo Clinic in Arizona Scottsdale, AZ, United States of America; 5 Biodesign Center for Innovations in Medicine, Arizona State University, Tempe, AZ, United States of America; 6 Department of Biochemistry and Molecular Biology, Mayo Clinic, Rochester, MN, United States of America; CIC bioGUNE, SPAIN

## Abstract

In the United States non-alcoholic fatty liver disease (NAFLD) is the most common form of chronic liver disease, affecting an estimated 80 to 100 million people. It occurs in every age group, but predominantly in people with risk factors such as obesity and type 2 diabetes. NAFLD is marked by fat accumulation in the liver leading to liver inflammation, which may lead to scarring and irreversible damage progressing to cirrhosis and liver failure. In animal models, genetic ablation of the protein G0S2 leads to alleviation of liver damage and insulin resistance in high fat diets. The research presented in this paper aims to aid in rational based drug design for the treatment of NAFLD by providing a pathway for a solution state NMR structure of G0S2. Here we describe the expression of G0S2 in an *E*. *coli* system from two different constructs, both of which are confirmed to be functionally active based on the ability to inhibit the activity of Adipose Triglyceride Lipase. In one of the constructs, preliminary NMR spectroscopy measurements show dominant alpha-helical characteristics as well as resonance assignments on the N-terminus of G0S2, allowing for further NMR work with this protein. Additionally, the characterization of G0S2 oligomers are outlined for both constructs, suggesting that G0S2 may defensively exist in a multimeric state to protect and potentially stabilize the small 104 amino acid protein within the cell. This information presented on the structure of G0S2 will further guide future development in the therapy for NAFLD.

## Introduction

Liver steatosis develops when these fatty acids become concentrated in liver tissue. Steatosis damages cells, resulting in large nutritional deficiencies, cell death, and permanent scarring of tissue by storage of triglycerides inside the cell [1]. Steatosis of liver tissue is associated with Non-Alcoholic Fatty Liver Disease (NAFLD) that is characterized by fat accumulation in the liver leading to liver inflammation, which may cause scarring and irreversible damage progressing to cirrhosis and liver failure. In the United States, NAFLD is the most common form of chronic liver disease, affecting an estimated 80 to 100 million people [2, 3]. It occurs in every age group, but especially in people with risk factors such as obesity and type 2 diabetes. The primary treatment for this disease is a liver section removal, exercise, lifestyle changes, and low fat diets [4–6]. To date, there are no drugs that have passed FDA standards in treatment of NAFLD, and thus the breaking down of triglycerides through lipolysis remains a primary target for drug development [7].

Before 2004, the only known enzyme that was responsible for lipolysis was hormone sensitive lipase. However, another lipase was suggested as mice with knockout mutations of hormone sensitive lipase still contained large amounts of the diglyceride breakdown product, indicating that the hydrolysis of triglycerides was still observed. Subsequent screens and further studies of adipose tissue led to the discovery of the rate limiting adipose triglyceride lipase (ATGL) for which G0S2 is also identified as one of the inhibitors [8–10]. When ATGL is inhibited by G0S2 lipids cannot be cleaved and lipids accumulate in the liver. G0S2 is a small protein of 11.4 kDa with a negative charge that binds strongly to ATGL, thus inhibiting the triglyceride hydrolase activity of ATGL. This claim was validated by a study in 2015 [11] with *G0S2* knockout mice, where the mice did not develop NAFLD despite being fed a high calorie diet and being severely obese. This study showed that G0S2 is a component for regulation in lipolysis, implicating it as potential target in the treatment of obesity and diabetes related conditions such as NAFLD [12–14]. Other studies have revealed several additional potential functions of G0S2 in lymphocyte activation, proliferation of leukemia cells, promotion of apoptosis, and reduction of tumor growth [15–19].

Future drug development aims to disrupt the ATGL-G0S2 interaction, with the goal to reach continuous activation of the triglyceride hydrolase activity in the presence of G0S2 in the cells to allow a breakdown of triglycerides in adipose [10]. The rational design of such drugs requires knowledge of the structures and the binding mechanism of G0S2 to ATGL and understanding of the function and dynamics of these two proteins and their functions for which structural information are required. However, there are no published structures, even partial, of the G0S2 or the ATGL protein. Here, we report the purification of a functional form of human G0S2. Further, we show that isotopically labeled G0S2 protein can be purified to sufficient amounts for NMR spectroscopy studies. The NMR studies revealed discernable chemical shifts for several of the residues of the N-terminal domain, but also indicated that parts of the protein are highly flexible as observed in a recent study where the G0S2 protein was found to be intrinsically disordered [20]. The results of this work will serve as a basis for further structural studies of G0S2 and the G0S2-ATGL interaction, providing significant information that helps develop future therapeutics for NAFLD treatment.

## Materials and methods

### Expression and purification of G0S2

#### Full length G0S2

The MBP-G0S2 construct containing the full-length open reading frame (ORF) of G0S2 was designed and generated using the following protocol. In brief, the ORF corresponding to mouse G0S2 (residues 1 to 104) was amplified and cloned into pET His6 MBP TEV LIC cloning vector (Addgene, catalog # 29708) and transformed into Lemo21(DE3) *E*. *coli* competent cells for protein expression (for DNA and protein sequences, see S1 Fig). Cells were grown in a starter 10-mL culture containing Lysogeny Broth (LB) medium consisting of 10 g/L tryptone, 5 g/L yeast extract, and 10 g/L NaCl with 0.1 mg/L ampicillin at 37°C for 10 hours. Cells from the starter culture were inoculated into a one liter LB also containing 0.1 mg/mL ampicillin and continued to grow until the optical density (OD) reached 0.6 absorbance at 600 nm. Expression of G0S2 in the cells was induced with 2 mM isopropyl B-D-1–thiogalactopyranoside (IPTG) and cells were grown for 4 hours at 37°C after induction. The cells were then harvested through centrifugation at 7000 x g for 10 minutes at 4°C and the cell pellet was frozen and stored at -80°C.

Protein purification of the MBP-G0S2 constructs was performed by lysis on 2.5 g of thawed harvested cell pellet degraded using 10 mg of lysozyme per 1 gram of wet cell in a buffer containing 20 mM Tris pH 7.5 and 100 mM NaCl on ice with an incubation at 4°C for 30 minutes. Following, the cells were mechanical lysed by sonication on ice using a Branson sonicator with 10-second/on and 30-second/off at 60% power for 10 pulses. The cell lysate was centrifuged at 36,000 x g for 30 minutes at 4°C for the purpose of removing insoluble cell debris. Following centrifugation, the lysate was then filtered through a 0.45 μm diameter nylon syringe filter and loaded onto a 10 mL amylose column containing high flow amylose resin (New England Biolabs). HPLC affinity chromatography was performed with a GE high pressure liquid chromatography system (HPLC). The column was pre-equilibrated with buffer A consisting of 20 mM Tris pH 7.5 and 300 mM NaCl. The column then was washed with buffer B containing 20 mM Tris pH 7.5, 300 mM NaCl and 40 mM maltose using a linear step gradient. MBP-G0S2 was eluted with buffer A plus 50 mM maltose. The eluted protein in buffer A was concentrated to 500 μL using a 10 kDa MWCO Amicon Ultra-15 centrifugal filter. A volume of 500 μL of the protein sample with a concentration approximately 15 mg/mL was further purified by being loaded on a Superdex 200 10/300 size exclusion column (SEC). The protein eluted from SEC was analyzed by 11% sodium dodecyl sulfate-polyacrylamide gel (SDS-PAGE) electrophoresis and developed by either Coomassie Blue staining or anti-His antibody immunoblotting to confirm the purity and presence of MBP-G0S2.

#### Truncated G0S2

**Fig 1:**
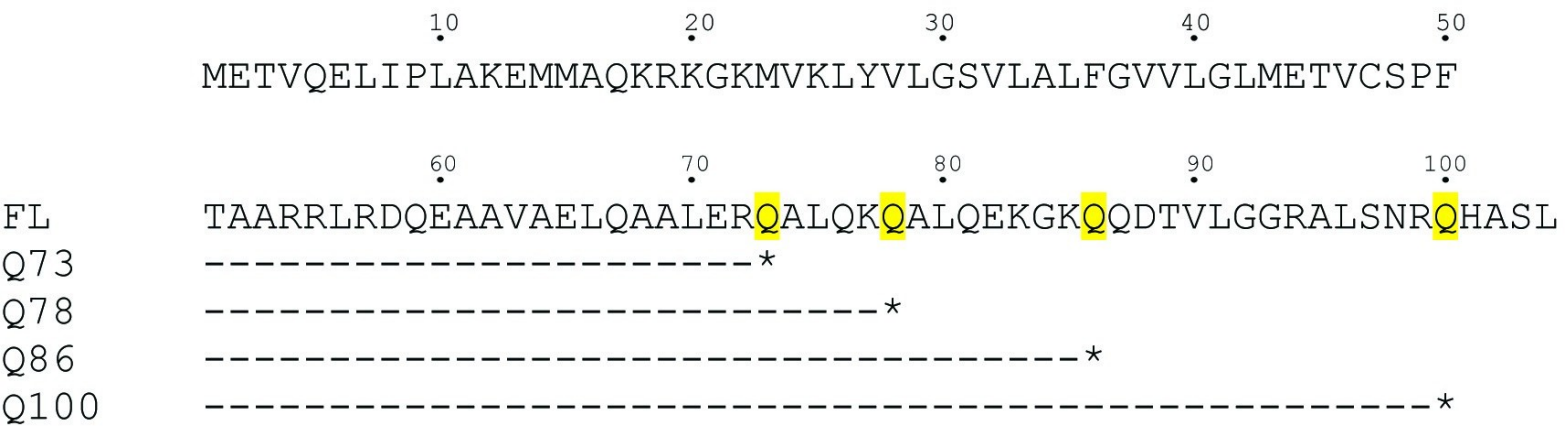
Truncation design of Q73, Q78, Q86, and Q100 compared to the full length (FL) MBP-G0S2. Nucleotides coding for glutamine at residues Q73, Q78, Q86, and Q100 were mutated to an ochre stop codon (shown as *) for the Q73, Q78, Q86, and Q100 constructs, respectively.

To confirm the formation of G0S2 oligomers, we generated 4 mutants, Q73, Q78, Q86, and Q100 using a point mutation on CAA encoded for glutamine (CAA) to the Ochre stop codon (TAA) that shorten the wild type G0S2 from 104 to 73,78, 86 and 100 residues from the full length construct (Fig 1). The naming convention for these mutants was created around the location of the mutated stop codon named Q73, Q78, Q86, and Q100. For the protein expression, the mutated genes were cloned into pET His6 Sumo TEV LIC plasmid (Addgene catalog # 29711) and transformed in Lemo21(DE3) *E*. *coli* cells (New England Biolabs). Cells were grown and protein purification were done as described for MBP-G0S2. The sample was then concentrated using a 50 kDa MWCO Amicon Ultra-15 centrifugal filter (Millipore). For the size exclusion run, a Superose 6 increase 10/300 column was used and the protein was eluted using buffer A.

#### SUMO-G0S2 monomers with and without ^13^C or/and ^15^N labeling

The *g0s2* gene was cloned into pET His6 Sumo TEV LIC plasmid (Addgene catalog # 29711) and transformed in Lemo21(DE3) *E*. *coli* cells (New England Biolabs).

For expression and purification SUMO-G0S2 (for DNA and protein sequences, see S2 Fig) without labeling, we followed the protocol described above for MBP-G0S2 with the exception of utilizing a reducing agent to remove oligomers. For purification of SUMO-G0S2 for 2D-NMR experiment, cells were grown in M9 media [21] containing isotopically labeled 4 g of ^13^C glucose or 1 g ^15^N ammonium chloride as described above per liter. After being induced with 2 mM IPTG at an OD_600_ of 0.6, cells were harvested after 4 hours past induction with incubation at 37°C by centrifugation at 7,000 x *g* at 4°C and stored at -80°C. Approximately 5 grams of wet cell pellet was thawed at 4°C for 10 minutes and resuspended in lysis buffer containing 20 mM Tris-HCl, pH 7.5, 1 mM 2-betamercaptoethanol (2-betamercaptoethanol) and 300 mM NaCl. Cells were lysed using a sonicator as described previously for G0S2. The lysate was centrifuged at 36,000 x *g* for 30 minutes at 4°C to remove unbroken cell debris.

The supernatant containing SUMO-G0S2 with ^13^C and ^15^N labeling was incubated with 5.0 mL of Talon Cobalt Resin (Prometheus Protein Biology) that was pre-equilibrated with the binding buffer C containing of 20 mM Tris pH 8.0, 300 mM NaCl, 2 mM 2-mercaptoethanol and 10 mM imidazole at 4°C for 20 minutes. The resin was loaded on a glass gravity column, washed with buffer C and eluted with buffer C containing 300 mM imidazole. The [^13^C,^15^N]-labeled SUMO-G0S2 was concentrated to 500 μL using an 10 kDa MWCO Amicon Ultra-15 centrifugal filter and loaded on a Superdex increase 200 column 10/300 (GE Healthcare) pre-equilibrated with 20 mM Tris-HCl pH 7.5, 1 mM 2-mercaptoethanol and 300 mM NaCl. The purified SUMO-G0S2 was concentrated to 5 mg/mL using a 10 kDa MWCO Amicon Ultra-15 centrifugal filter. The fractions collected from the last purification step were analyzed by SDS-PAGE gel and visualized by Coomassie Blue staining and the use of 5 μL precision plus protein standard (Bio-Rad). For both constructs we attempted to cleave the MBP and SUMO fusion tags from the G0S2 protein through the TEV protease as well as the SUMO protease, but the cleavage of the tag led to instability in solution and precipitation of G0S2 both at room temperature and at 4C for both constructs. We tried different ways to stabilize the G0S2 protein during cleavage including variation of the ionic strength and pH and addition of detergent but the problem persisted and we therefore continued our studied with the non-cleaved proteins.

### Determination of G0S2 oligomeric hydroxamic radius by dynamic light scattering (DLS) and Transmission Electron Microscopy (TEM)

A volume of 5 μL of MBP-G0S2 at 5 mg/mL was used for DLS measurement. The sample was placed on a 22 mm silicon glass cover slide (Hampton Research) and positioned as a hanging drop on a 24-well crystallization plate (VDX, Hampton Research). The sample was then brought into the pathway of a 750 nm laser of a Spectro Size 302 DLS instrument (Molecular Dimensions) at a 40 degree scattering angle at room temperature. The hydrodynamic radius and polydispersity index (PDI) were estimated from the instrument software. For TEM imaging, MBP-G0S2 at 0.003 mg/mL was placed on a mesh copper grid. The sample was visualized under the Phillips CM 12 transmission electron microscope and several images at 140,000X were taken.

### Small Angle X-ray Scattering (SAXS)

Small angle x-ray scattering (SAXS) and multi angle light scattering (MALS) experiments were performed at the Argonne National Laboratory Advanced Photon Source. The MBP-G0S2 sample at a concentration of 6 mg/mL in buffer A was injected onto the HPLC at the 18-ID-D SAXS beamline 1 at a rate of 0.75 mL/min utilizing a sample delivery system that included an inline size exclusion multi angle light scattering(SEC-MALS) device with quasi elastic light scattering capabilities. For the SAXS experiment, a small angle camera was introduced to a beam line that utilized a 150 μm x 50 μm height by volume beam size. The flux of the beam line was 2 x 10^13^ photons per second at 12 KeV and the scattered radiation was detected and collected with a Pilatus 3S 1M detector with a sample distance of 3 meters. SAXS data reduction was done by using the ATSAS data analysis software through PRIMUS program [22]. Further data analysis was done by indirect Fourier transformation by GNOM software [23] to obtain un-smeared SAXS curves, which then was used to fit in the data of known protein shapes to obtain a low-resolution model.

### Lipase inhibitory activity assay

HeLa cells were transfected with an ATGL expressing plasmid (pRK-ATGL) in a 10-cm dish using Lipofectamine 2000 overnight and lysed on ice by sonication in 1.0 mL of a lysis buffer (0.25 M sucrose, 1 mM EDTA, 1 mM Tris-HCl pH 7.4, 1 mM dithiothreitol, 20 μg/mL leupeptin, 2 μg/mL antipain and 1 μg/mL pepstatine). The cell lysate was clarified by centrifugation at 15,000 x *g* for 10 minutes at 4°C. The supernatant containing ATGL was used as the lipase source for the triglyceride hydrolase activity assay.

Triglyceride hydrolase activity assay was carried out with the tagged G0S2 proteins using a lipid emulsion labeled with [9,10-^3^H]-triolein as the reaction substrate. Briefly, an 80 μL of 2 μg and 10 μg of purified MBP-G0S2 or SUMO-G0S2 was added to an 80 μL reaction mix containing 40 μL of lysis buffer and 40 μL supernatant from Hela cell lysate. The G0S2/ATGL mixture was incubated with 80 μL of substrate solution for 60 min at 37°C. Reactions were terminated by adding 2.6 mL of methanol/chloroform/heptane (10:9:7,vol/vol/vol), 0.84 mL of 0.1 M potassium carbonate, and 0.1 M boric acid at pH 10.5. Following centrifugation at 800 x *g* for 15 min, radiolabeled fatty acids in 1 mL of upper phase were measured by liquid scintillation counting. The triglyceride hydrolase activity was determined by number of disintegration per minute (DPM) of [9,10-^3^H]-triolein after hydrolysis by ATGL using a scintillation counter.

### Nuclear magnetic resonance spectroscopy of SUMO-G0S2 labeled with ^13^C or/and ^15^N

NMR data were collected at 25.0°C on a Bruker 850 MHz spectrometer utilizing a 5mm TCI Cryoprobe at the Magnetic Resonance Research Center at Arizona State University. NMR spectra of SUMO-G0S2 were obtained by carrying out a series of 2D- and 3D-NMR experiments including ^15^N-HSQC, HNCA, HNCO, HNCACB, CBCA(CO)NH, CCONH, HN(CO)CA, HCCH-TOCSY, and NOESY-^15^N-HSQC [24–30]. The HCCH-TOCSY experiment and the NOESY-^15^N-HSQC experiment were done with 60 ms and 120 ms mixing times respectively. The 2D-NMR experiments were carried out using SUMO-G0S2 labeled with ^15^N whereas the 3D-NMR experiments were performed using SUMO-G0S labeled with ^13^C, and ^15^N. Data were processed with mddNMR [31–34] NMRPipe [35] and analyzed using the CCPNMR analysis software [36].

## Results

### Purification of MBP-G0S2 and SUMO-G0S2

**Fig 2:**
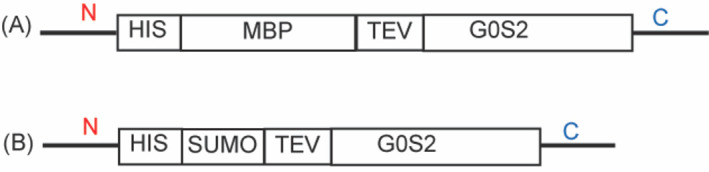
G0S2 construct design. MBP-G0S2 construct (A) contains 495 residues (54 kDa) whereas SUMO-G0S2 (B) is shorter with 214 residues (22.5 kDa). Both full length MBP-G0S2 and SUMO-G0S2 contain an N-terminal 6xHis-tag and a TEV cleavage site (ENLYFQ*S, where * indicates the cleavage site). Abbreviations: MBP, maltose-binding protein; SUMO, small ubiquitin-like modifier; TEV, Tobacco Etch Virus.

**Fig 3:**
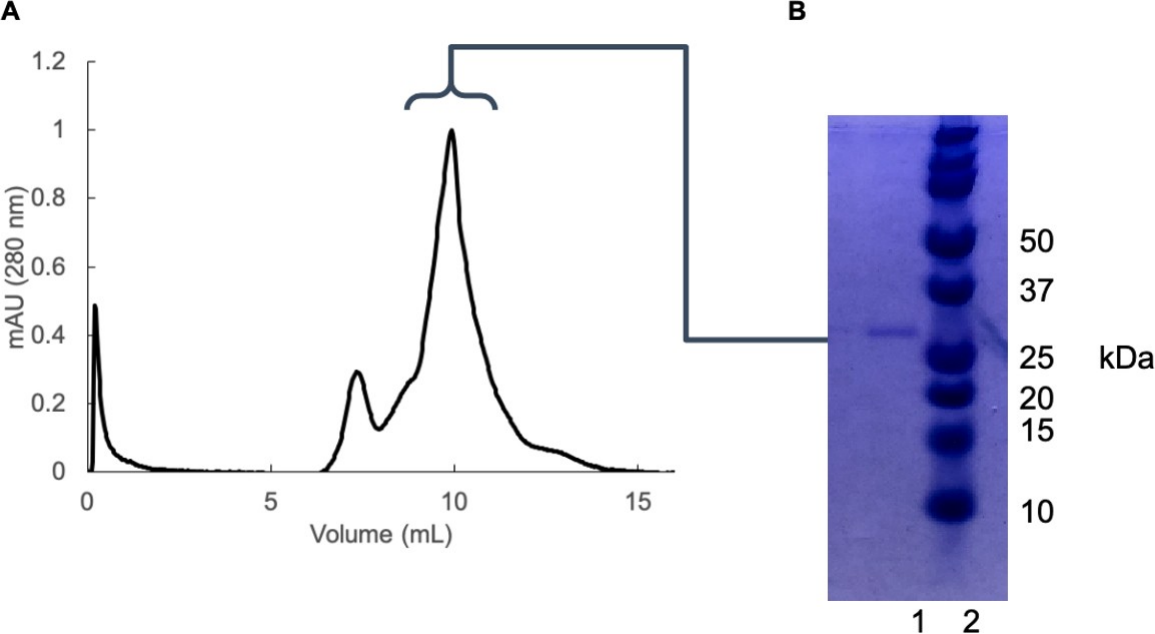
Purification of ^13^C,^15^N-labeled SUMO-G0S2 for NMR structural determination. (A) Size exclusion chromatogram of ^13^C,^15^N-labeled SUMO-G0S2 showed the protein eluted at 11 mL, indicated by the bracket above, where the full width half maximum (FWHM) of the peak was pooled for the Coomassie gel. (B) Coomassie blue stained SDS-PAGE of ^13^C ^15^N-labeled SUMO-G0S2 after SEC. *Lane 1*: Approximately 1 μg of SUMO-G0S2 from peak 2 shown on (A). *Lane 2*: molecular marker.

Two constructs were used for the structural studies in this paper: a maltose binding protein (MBP) construct (Fig 2A) and a SUMO-G0S2 construct (Fig 2B). The rationale for the use of these two constructs was that they were screened from a long list of produced constructs and these two, MBP-G0S2 and SUMO-G0S2, were successfully expressed in the *E*.*coli* expression system and purified to high homogeneity at yields of around 5 mg for 1 liter of culture. For MBP-G0S2, the protein was purified by amylose affinity chromatography, following by a SEC. For SUMO-G0S2, the protein was purified by immobilized metal affinity chromatography following by SEC (Fig 3A). The His-tag was included in both constructs due to protein aggregation once the tag was removed. Both for the SUMO-G0S2 and MBP-G0S2 we have tried to cleave the large soluble tags after purification. However through cleavage by the TEV protease as well as usage of a SUMO protease, the G0S2 protein became unstable and precipitated upon cleavage of the tags so the G0S2 protein by itself could not be stabilized for structural and functional studies through a lipase inhibitory assay. As we were able to show that the tagged proteins were fully functionally active, we continued our studies with the tagged G0S2 proteins. Fig 3B shows SUMO-G0S2 migrated on the Coomassie-stained SDS-PAGE gel at the apparent molecular weight of 27 kDa, that is consistent with the predicted molecular weight of SUMO-G0S2. A negligible amount of contaminants was shown on the gel confirming the sample was highly homogenous (Fig 3B). In addition, MBP-G0S2 was also purified (S2A and S3 Figs) to high homogeneity as shown in lane 2 of S3B Fig where a lane is visible at the apparent weight of 54 kDa, the predicted weight of MBP-G0S2. To investigate the formation of MBP-G0S2 oligomers, we obtained transmission electron microscopy images of MBP-G0S2 particles, measured dynamic light scattering, and performed small angle X-ray scattering of MBP-G0S2 and MBP-G0S2 truncated mutants. S5 Fig shows the TEM images of MBP-G0S2 with an estimated average size of approximately 15 nm to observe dependence on G0S2 in the creation of oligomers.

A long decay time in dynamic light scattering experiments is typical of large particles. A decay time of approximately 1 msec was observed in the DLS correlogram (S6A Fig) that indicated the presence of a large complex. Furthermore, the DLS radius frequency histogram estimated the hydrodynamic radius of the complex was approximately 20 nm (S6B Fig). The result from SAXS measurement further suggests the formation of an oligomer and the presence of a large hollow interior when the scatter data from SAXS as the shape of the slope suggests a hollow, ordered structure (S6C Fig). To determine if the complex formation was due to interaction of residues on G0S2, we measured the particle sizes of the truncated MBP-G0S2 constructs Q86, Q78, and Q73, of which the amino acid sequence is shorter than the full length G0S2 (S6D Fig). The DLS results showed the radius decreased to 16 nm, 13 nm, and 12 nm for Q86, Q78, and Q73, respectively, suggesting that G0S2 oligomer formation was due to interactions between G0S2 residues. This was then further confirmed through the DLS results which were done on SUMO-G0S2, where the large oligomer still formed showing a radius of 16 nm (S7A and S7B Fig).

### Purified tagged G0S2 inhibits the activity of ATGL

**Fig 4:**
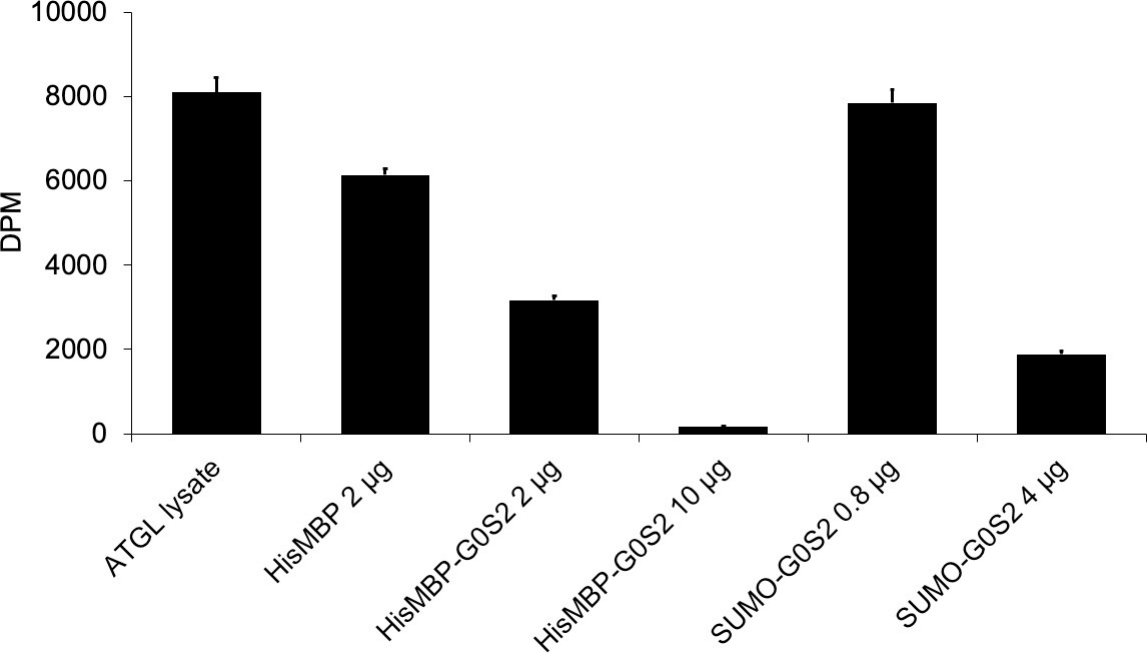
MBP-G0S2 and SUMO-G0S2 inhibit triglyceride hydrolase activity of ATGL. Inhibitory activity assay of HisMBP-G0S2 and SUMO-G0S2 was determined using a lipid emulsion labeled contain [9, 10-^3^H]-triolein as substrate and measured by liquid scintillation counting measured by disintegrations per minute (DPM) and error bars represent percent error. Both constructs were purified through size exclusion chromatography.

Following purification, Fig 4 shows the results of G0S2 inhibition activity on ATGL when [9,10-^3^H]-triolein was used as a substrate. The larger DPM or disintegrations per minute were observed, the more radiolabeled fatty acids were in the sample due to the breaking down of [9,10-^3^H]-triolein by ATGL. The *E*. *coli* expressed ATGL lysate that was unpurified, which has been unable to be purified past this point, and HisMBP, which did not contain G0S2, were used as the controls for no inhibition. All activity assays were performed with the non-cleaved MPP-G0S2 and non-cleaved SUMO-G0S2 proteins. Fig 4 shows the sample “ATGL lysate”, which contains only [9,10-^3^H]-triolein and ATGL lysate, displayed the highest intensity because the triglyceride was broken down into radiolabeled fatty acids. Similarly, the DPM value was high when 10 μg of HisMBP protein was added to [9,10-^3^H]-triolein and ATGL reaction, which indicates triglyceride hydrolase activity in the lysate. However, when replacing 2 μg HisMBP by 2 μg of MBP-G0S2, the DPM value decreased approximately 60% compared to the HisMBP confirming less radiolabeled fatty acids were present in the sample. The DPM value decreased even more when 4 μg of SUMO-G0S2 was used in the reaction. Regardless if MBP-G0S2 or SUMO-G0S2 were used, the number of DPM counts was concentration dependent as smaller DPM value was obtained for 10 μg compared to 2 μg MBP-G0S2, and 4 μg compared to 0.8 μg SUMO-G0S2. These results demonstrate that each of the purified constructs was functional in terms of inhibiting ATGL hydrolase activity.

### NMR spectroscopy of SUMO-G0S2

**Fig 5:**
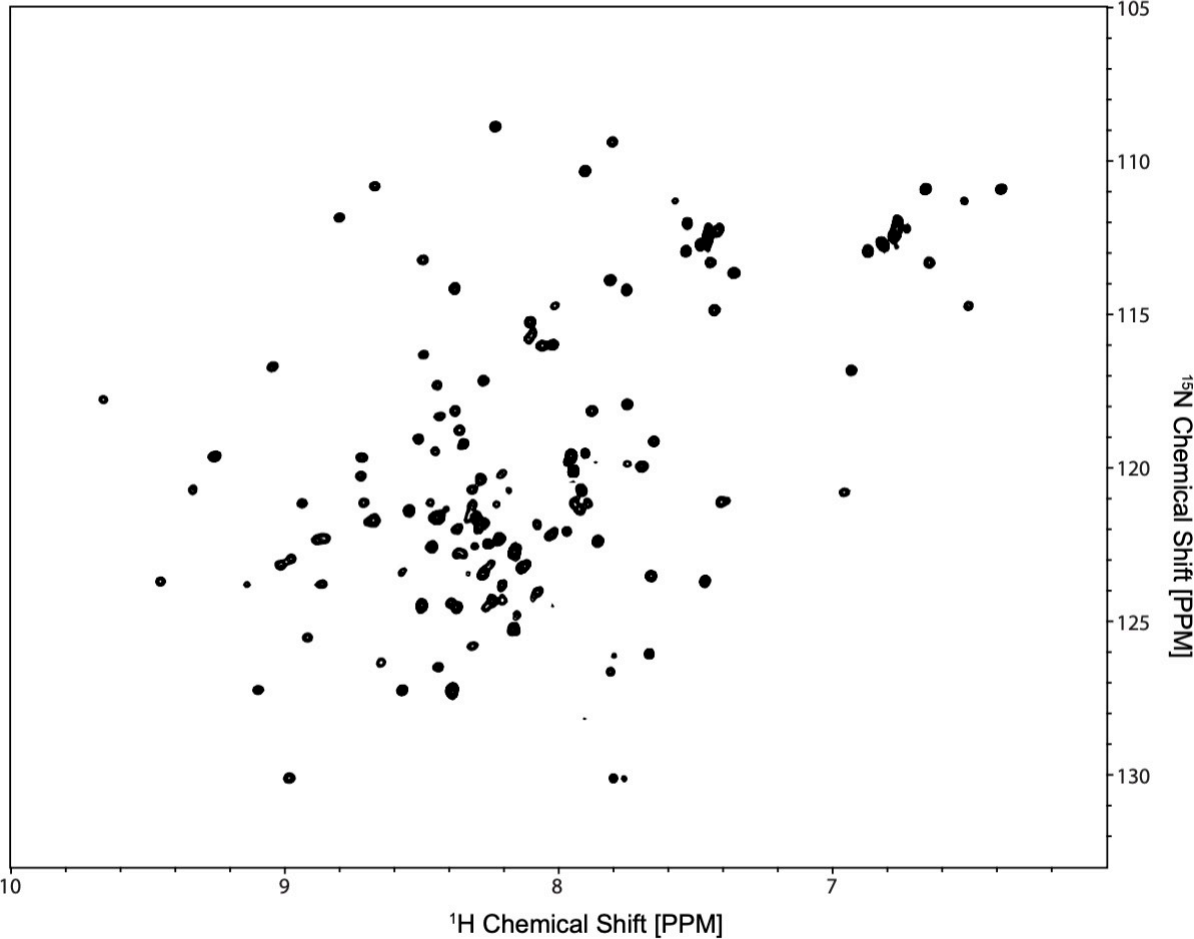
SUMO-G0S2 spectra. ^15^NHSQC spectra from nitrogen-labeled SUMO-G0S2.

Following confirmation of activity, a monomeric ^15^N-labeled SUMO-G0S2 sample was prepared and a ^15^NHSQC NMR measurement was performed on a 600 MHz spectrometer. Analysis of the SUMO-G0S2 ^15^NHSQC spectrum (Fig 5) when compared to a purified SUMO ^1^N^15^HSQC [37] displayed a shift of existing peaks and an increase in number of peaks, but not enough for the SUMO-G0S2 construct. The data provided evidence of a well dispersed sample (Fig 5) and similarities to the published SUMO structures ^15^NHSQC spectra [37]. To further analyze the spectra, three-dimensional data sets consisting of the HNCA, HNCO, HNCACB, CBCA(CO)NH, CCONH, HN(CO)CA, HCCH-TOCSY, and NOESY-^15^NHSQC, were used to aid in the assignment of the SUMO-G0S2 protein.

**Fig 6:**
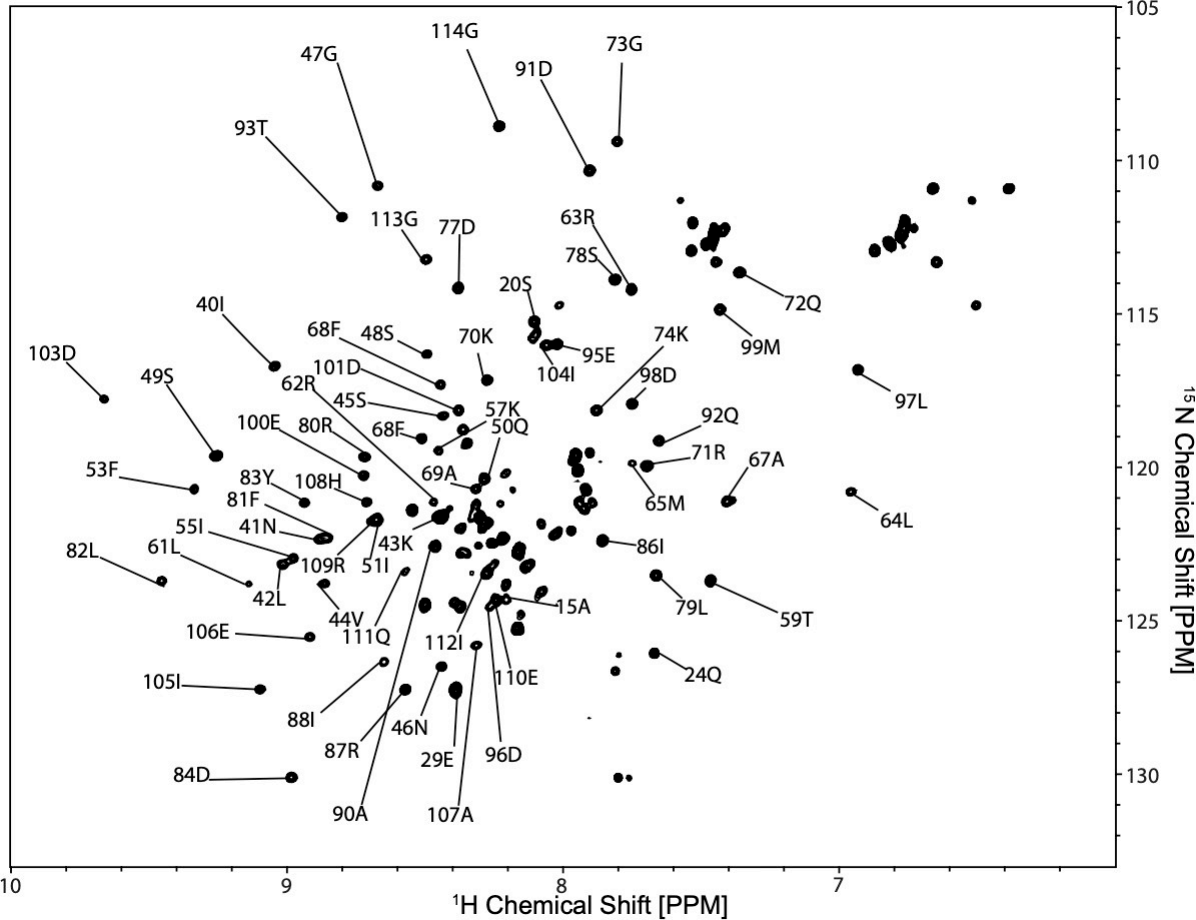
SUMO-G0S2 spectra. ^15^NHSQC spectra from nitrogen-labeled SUMO-G0S2, with SUMO assigned resonances.

**Fig 7:**
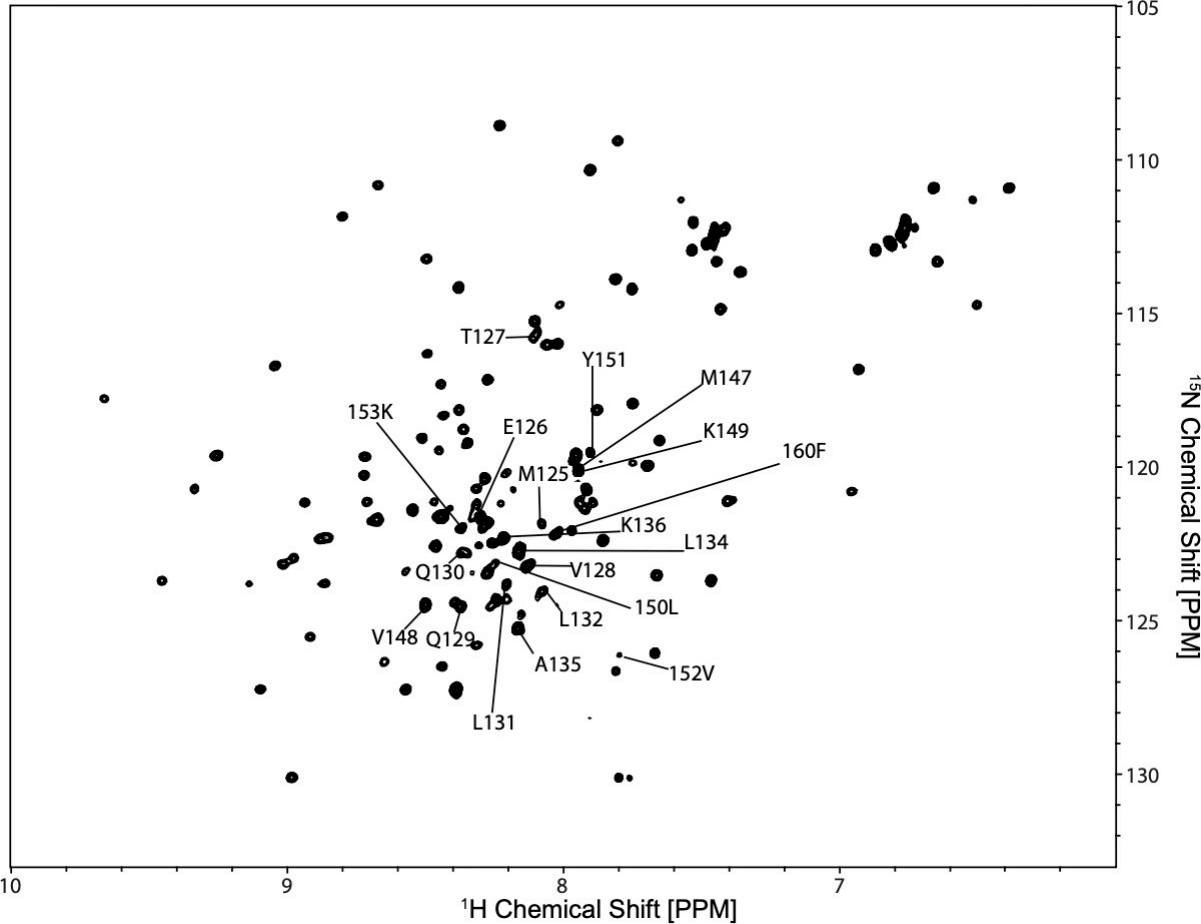
SUMO-G0S2 spectra. ^15^NHSQC spectra from nitrogen-labeled SUMO-G0S2 and identification of the assigned G0S2 residues.

Utilizing collected spectra on the alpha, beta, and carbonyl carbons and probability values to assign these resonances, large sections of the SUMO protein were assigned. These assignments (Fig 6) are consistent with the published data from the 2002 solution structure of the SUMO protein [37]. Following, the unassigned resonances were examined and the presence of small segments of the G0S2 protein near the N-terminus was confirmed by observing a chain of sequential alpha and beta carbons (S8 Fig). A small other segment of the protein was observed starting at Methionine 147 to Lysine 153, these assignments can be seen in Fig 7.

## Discussion

Currently, no structure of G0S2 have been determined. This research aims to aid in the discovery of developing a drug for treatment of NAFLD by obtaining a high-resolution structure of the 11 kDa G0S2 protein by NMR spectroscopy. We have optimized a protocol for the expression and purification of two different G0S2 constructs MBP-G0S2 and SUMO-G0S2. Both constructs were expressed and purified with sufficient quantities for NMR analysis. In addition, both constructs were functionally active as shown in Fig 4. The polydispersity index (PDI) is defined as a ratio of molecular weight average and number average molecular weights providing the range of molecular weight distribution of molecules in a solution. The PDI values of MBP-G0S2 and SUMO-G0S2, suggested that the protein is mostly distributed in large complexes; however, it was not monodisperse enough to pursue structure studies by cryoelectron microscopy (S4D Fig). The large oligomeric complexes described in this research provide insight on how this small protein could survive in the body. By breaking up the SUMO-G0S2 oligomer through use of a reducing agent, the 26 kDa SUMO-G0S2 construct was stable and used for two- and three-dimensional NMR experiments at room temperature. These preliminary NMR data support that the purified protein are suitable for further NMR structure work.

Although atomic resolution structure of G0S2 has not yet been determined it is predicted to contain two alpha helices separated by a hydrophobic sequence and a beta sheet [14, 38]. One of the major challenges of studying this protein from a structural standpoint is the disordered hydrophobic predicted structure near the C-terminus of the construct when confirmed in programs such as XTALPRED [14, 39]. To overcome protein aggregation due to this disordered region, in this study we fused a small ubiquitin-like modifier(SUMO) tag to the N-terminus of the G0S2 protein, with the aim of obtaining a soluble form of G0S2 and trying to stabilize the structured region of the G0S2 protein during and after purification for NMR measurements [40]. Moving forward, several NMR experiments were performed to determine more information on the structure of SUMO-G0S2. The G0S2 protein contains a small hydrophobic region that is partially found in the obtained ^15^NHSQC that is responsible for the binding and inhibition of ATGL in lipolysis by competing for an active site in ATGL with a protein known as CGI-58 that is responsible for the activation of ATGL [9]. A small peptide containing Tyr27-Met43 of G0S2, designed and expressed in *E*. *coli* expression system, was found to be a functional inhibitor of ATGL [13].

We predict that with further analysis of obtained spectra and more NMR experimentation, a structure can be calculated from the hydrophobic domain as well at the N-terminus where portions of the data have been collected; specifically in the first 40 residues of the G0S2 protein. To solve the structure of this protein, more NMR data needs to be obtained on the residues Tyr27-Met43. A number of missing or overlapping residues was observed in the SUMO-G0S2 NMR spectrum around the intrinsically disordered region (Fig 5) [20], which is consistent with previous prediction [20, 41]. This problem can be overcome by conducting CON series of experiments [42] in addition to the collected spectrum as shown in S5 Fig. The combination of results from current measurements and CON series of experiments can lead to more reliable residue assignments by taking advantage of the slow relaxation and the large chemical shift dispersion for analyzing these experimental results. Another potential solution to the data acquisition problem would be to collect a completely deuterated set of TROSY experiments, which would provide high resolution proton data to supplement the existing data sets [43] or addition of ATGL to stabilize this protein. For the future work, we will perform a set of CON experiments to complete assignments of the functional domain of G0S2, as well as working with a SUMO-G0S2 construct containing only the functional domain of G0S2. Additionally, the idea of studying this protein in a membrane environment will be pursued through reconstitution of the G0S2 constructs in the presence of lipid nano disc. Further obtained structural information of this protein and its interactions with ATGL will aid in the future development of drugs to inhibit the G0S2 proteins binding to adipose triglyceride lipase and create a possible effective therapy against NAFLD.

## Supporting information

S1 Fig Sequences for plasmid pHis6-MBP-g0s2, for expression of MBP-G0S2. (**A**) DNA sequence. (**B**) Protein sequence.
(ZIP)

S2 Fig Sequences for plasmid pHis6-SUMO-g0s2, for expression of SUMO-G0S2. (**A**) DNA sequence. (**B**) Protein sequence.
(ZIP)

S3 Fig Affinity chromatography of MBP-G0S2. Shown is the absorbance trace and percent Buffer B (20 mM Tris pH 7.5, 300 mM NaCl, and 40 mM maltose) in amylose affinity chromatography. Bracket indicates peak where the full width half maximum was eluted for further purification.
(TIF)

S4 Fig Purification of MBP-G0S2. (A) SEC chromatogram of MBP-G0S2. The protein was purified using a Superose 6 increase 10/300GL SEC column. MBP-G0S2 eluted at 15-mL eluent. (B) Coomassie blue stained SDS-PAGE gel of MBP-G0S2. *lane 1*: ladder, *lane 2*: purified MBP-G0S2 by nickel Immobilized Metal Affinity Chromatography (IMAC) and size exclusion chromatography.
(TIF)

S5 Fig TEM Images of MBP-G0S2. TEM image of 0.003 mg/mL MBP-G0S2 purified sample, purified the same way as Fig 3A, on mesh copper grid.
(TIF)

S6 Fig Dynamic light scattering measurement of MBP-G0S2 and the truncated MBP-G0S2. (A) DLS correlogram of all MBP-G0S2 and the truncations. (B) DLS frequency histogram of the protein radius versus particle distribution. (C) Scatter plot from SAXS analysis of MBP-G0S2 shows the log of the experimental intensity is plotted versus reciprocal size. (D) Table summary of MBP-G0S2 and the truncated MBP-G0S2 proteins DLS data.
(TIF)

S7 Fig Dynamic light scattering measurement of SUMO-G0S2. (A) DLS correlogram of SUMO-G0S2, (B) DLS frequency histogram of the protein radius versus particle distribution.
(TIF)

S8 Fig Sequence of alpha and beta carbons of SUMO-G0S2. Display of G0S2 residues starting at 127 in the SUMO-G0S2 protein sequence and what missing residues are needed for functionality and for the functionality domain. Experiments are shown in triplets for each amino acid where the first experiment is the CC(CO)NH where carbons are shown in purple. The next experiment is the CBCA(CO)NH where the carbons are shown in blue, and the HNCACB where the alpha carbons are shown in red and the beta carbons are shown in black.
(TIF)

S1 File Part 2 of data included for the publication consist of 15NHSQC spectra for two-dimensional analysis as well as the HNCA, HNCA, HNCACB, CBCA(CO)NH, CONCH, HN(CO)CA, HCCH-TOCSY, and NOESY15NHSQC three dimensional experiments. Data is included in zip files in format for CCPNMR software.
(ZIP)

S2 File Part 1 of data included for the publication consist of 15NHSQC spectra for two-dimensional analysis as well as the HNCA, HNCA, HNCACB, CBCA(CO)NH, CONCH, HN(CO)CA, HCCH-TOCSY, and NOESY15NHSQC three dimensional experiments. Data is included in zip files in format for CCPNMR software.
(ZIP)

S1 Raw image Raw images of Coomassie blue stained SDS-PAGE gels of SUMO-G0S2 and MBP-G0S2 both purified by affinity and size exclusion chromatography. (TIF)
