# Supplementary figures and images for: Biophysical characterization and a roadmap towards the NMR solution structure of G0S2, a key enzyme in non-alcoholic fatty liver disease
Source: PLoS One. 2021 Jul 14;16(7):e0249164. doi: 10.1371/journal.pone.0249164 (PMC8279337; doi:10.1371/journal.pone.0249164)

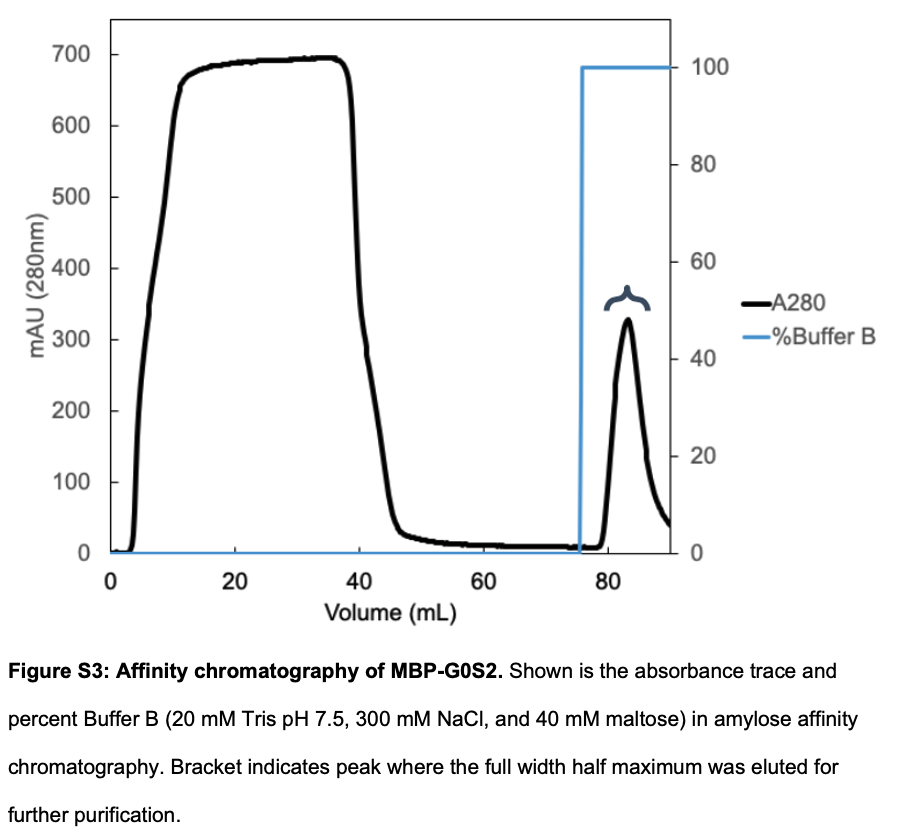

Supplement: S3 Fig — Shown is the absorbance trace and percent Buffer B (20 mM Tris pH 7.5, 300 mM NaCl, and 40 mM maltose) in amylose affinity chromatography. Bracket indicates peak where the full width half maximum was eluted for further purification. (TIF) [file pone.0249164.s003.tif]

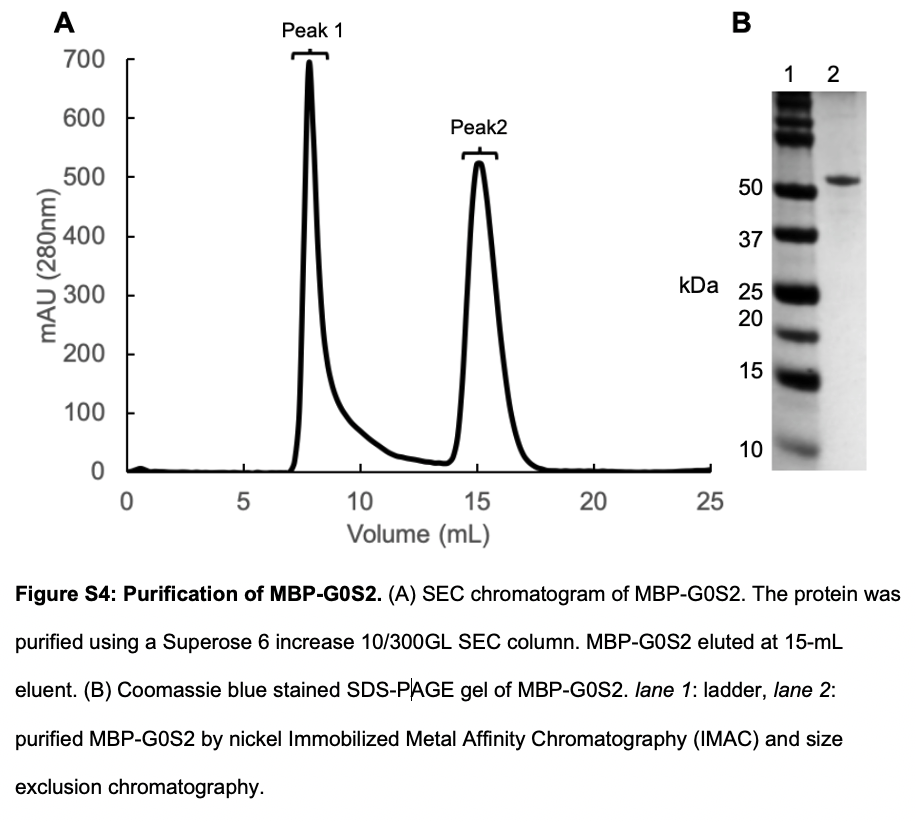

Supplement: S4 Fig — (A) SEC chromatogram of MBP-G0S2. The protein was purified using a Superose 6 increase 10/300GL SEC column. MBP-G0S2 eluted at 15-mL eluent. (B) Coomassie blue stained SDS-PAGE gel of MBP-G0S2. lane 1: ladder, lane 2: purified MBP-G0S2 by nickel Immobilized Metal Affinity Chromatography (IMAC) and size exclusion chromatography. (TIF) [file pone.0249164.s004.tif]

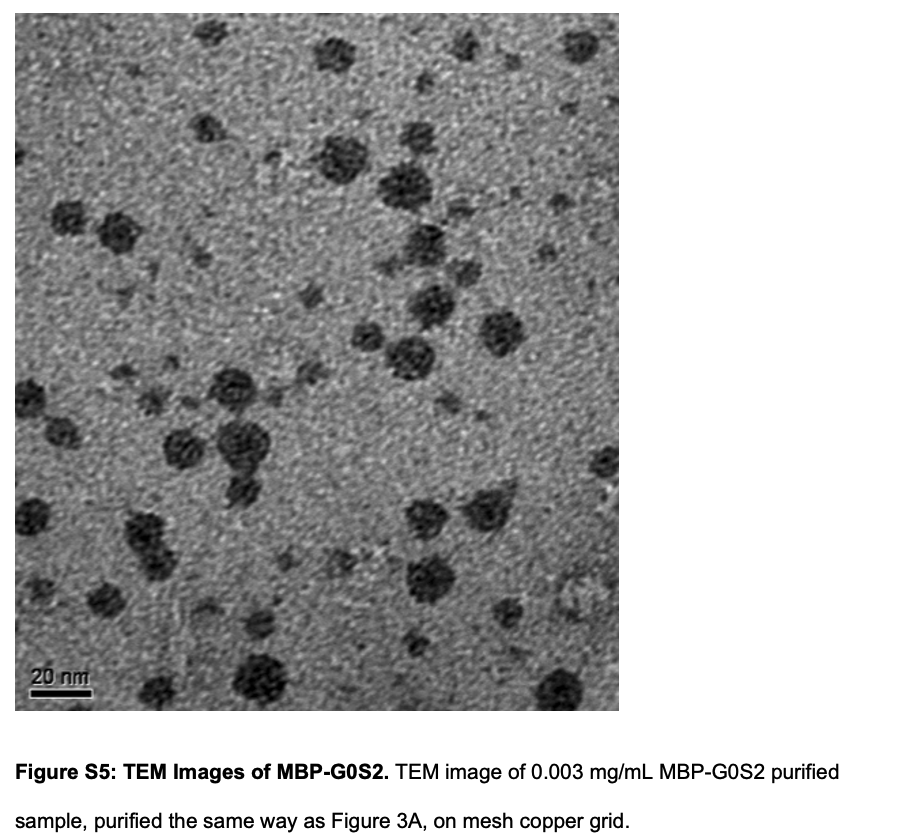

Supplement: S5 Fig — TEM image of 0.003 mg/mL MBP-G0S2 purified sample, purified the same way as Fig 3A, on mesh copper grid. (TIF) [file pone.0249164.s005.tif]

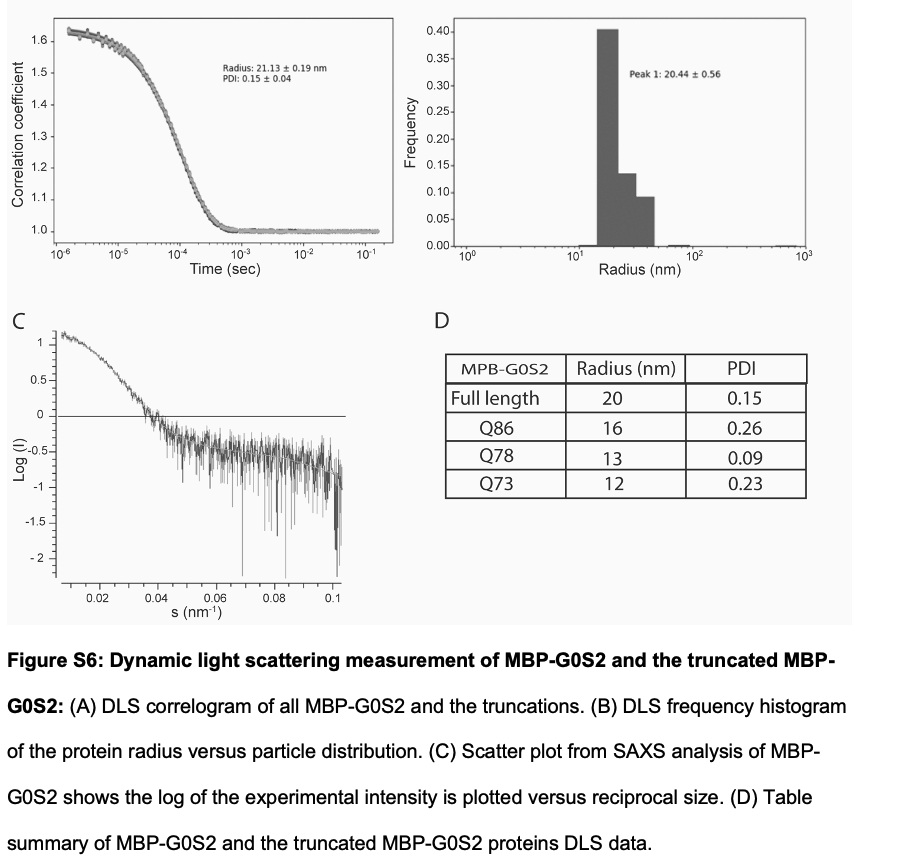

Supplement: S6 Fig — (A) DLS correlogram of all MBP-G0S2 and the truncations. (B) DLS frequency histogram of the protein radius versus particle distribution. (C) Scatter plot from SAXS analysis of MBP-G0S2 shows the log of the experimental intensity is plotted versus reciprocal size. (D) Table summary of MBP-G0S2 and the truncated MBP-G0S2 proteins DLS data. (TIF) [file pone.0249164.s006.tif]

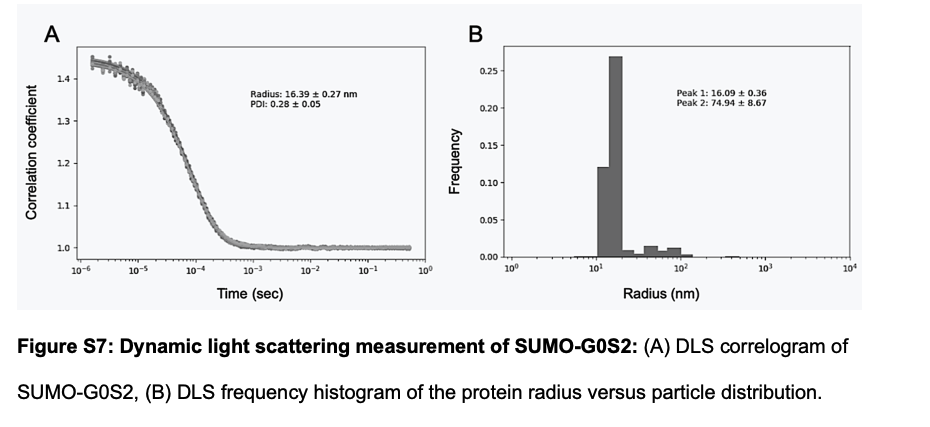

Supplement: S7 Fig — (A) DLS correlogram of SUMO-G0S2, (B) DLS frequency histogram of the protein radius versus particle distribution. (TIF) [file pone.0249164.s007.tif]

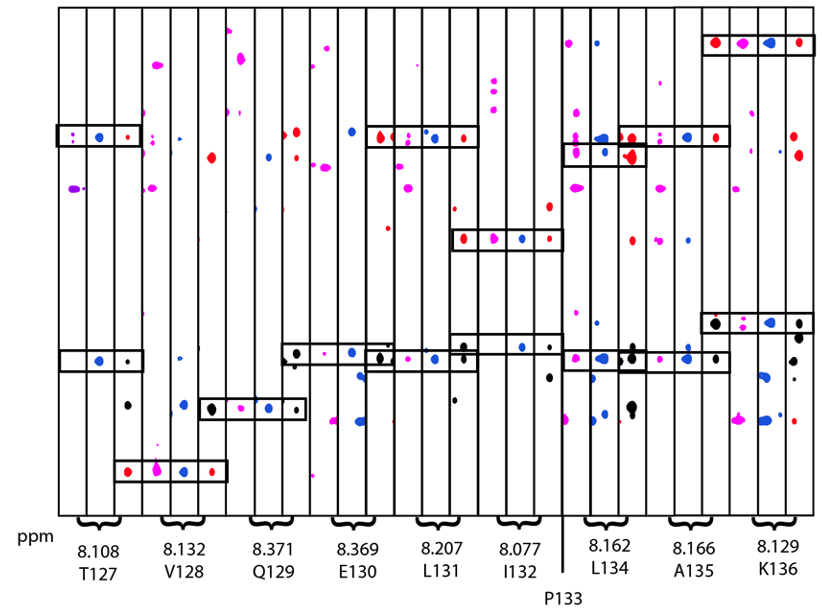

Supplement: S8 Fig — Display of G0S2 residues starting at 127 in the SUMO-G0S2 protein sequence and what missing residues are needed for functionality and for the functionality domain. Experiments are shown in triplets for each amino acid where the first experiment is the CC(CO)NH where carbons are shown in purple. The next experiment is the CBCA(CO)NH where the carbons are shown in blue, and the HNCACB where the alpha carbons are shown in red and the beta carbons are shown in black. (TIF) [file pone.0249164.s008.tif]

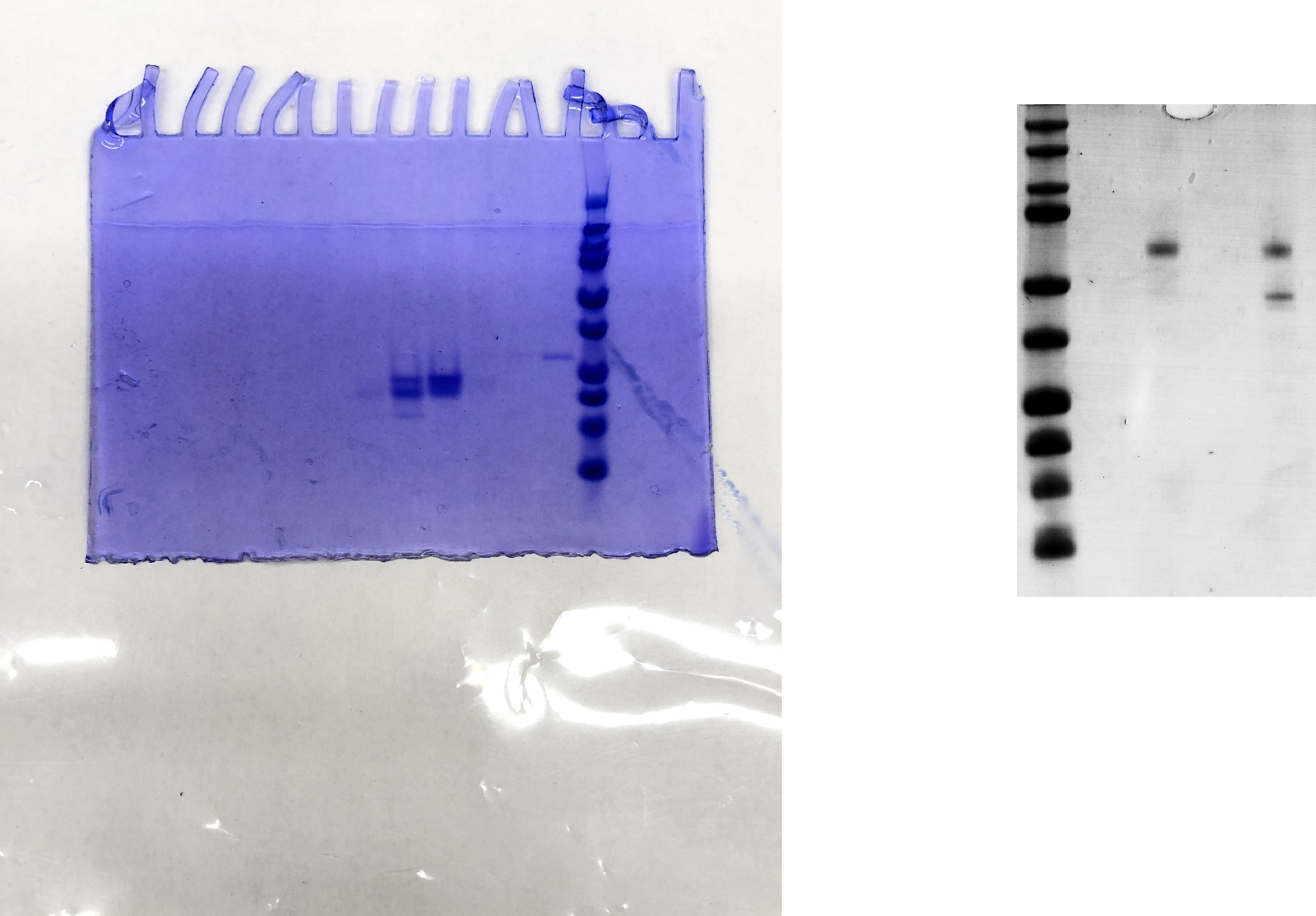

Supplement: S1 Raw image — (TIF) [file pone.0249164.s011.tif]
